# Incidence and predictors of Adverse Drug Reaction (ADR) among adult HIV positive patients on anti-retroviral treatment in Arba Minch town public health facilities, southern Ethiopia: A retrospective cohort study, 2020

**DOI:** 10.1371/journal.pone.0251763

**Published:** 2021-05-27

**Authors:** Abdulbasit Sherfa, Dereje Haile, Menaye Yihune, Sewenet Sako

**Affiliations:** 1 Wolaita Zone Health Department, School of Public Health, College of Medicine and Health Science, Wolaita Sodo University, Sodo, Ethiopia; 2 Reproductive Health and Nutrition Department, School of Public Health, College of Medicine and Health Science, Wolaita Sodo University, Sodo, Ethiopia; 3 School of Public Health, College of Medicine and Health Science, Arba Minch University, Arba Minch, Ethiopia; University of Mississippi Medical Center, UNITED STATES

## Abstract

**Background:**

Besides its contribution in the treatment of Human Immunodeficiency Virus-infected patients, anti-retroviral drugs may also cause mild to serious adverse effects. It is the main causes for poor drug adherence, treatment discontinuation and changes in Anti-Retroviral Treatment (ART) regimens. Thus, the aim of this study was to assess the incidence and predictors of adverse drug reaction among adult Human Immunodeficiency Virus positive patients on anti-retroviral treatment from January 1, 2013 up to December 30, 2018 at Arba Minch town public health facilities, Southern Ethiopia.

**Methods:**

An institution based retrospective cohort study was conducted on 456 charts. Secondary data was collected by structured questionnaire. Data were entered in Epi-data version 4.4.2 software and exported to STATA Version 14 for analysis. The Kaplan-Meier survival curve with Log-rank was used to estimate survival time. Bi-variable and multivariable Cox proportional hazard regression models were fitted to identify predictors of adverse drug reaction. In the bi-variable analysis, the variables with p-value < 0.25 were eligible for multivariable analysis. Finally, variables with p-values less than 0.05 in the multivariable Cox regression were considered as independent predictors. The statistical significance was declared at p-value<0.05.

**Results:**

Out of 456 patients observed for 14,903 person-months (pm), 79 experienced adverse drug reactions and the overall incidence density rate was 0.53/100 pm, CI: 0.42–0.66/100 person’s month or 6.36 per 100 Pearson year of observation. Females were at higher risk of experiencing adverse drug reactions (ADRs) compared to males [AHR = 2.35; CI (1.18, 4.69)]. The risk of experiencing ADRs among patient with advanced WHO clinical stage (stage III and IV) was higher compared to stage I and II [AHR = 3.0, CI (1.22, 7.37)]. The risk of experiencing an ADR was at any given time higher among AZT and NVP containing regimens compared to TDF and EFV containing regimens. Finally, the risk of ADR among those patients from the health center was reduced by 59% compared to hospital patients, [AHR = 0.41, CI (0.17, 0.97)].

**Conclusion and recommendation:**

The incidence rate of ADRs was reduced among patients on ARVs compared to previous studies and it was high during the early years of ART initiation. HIV patients should be closely followed in the early years of ART initiation, since this is the time of highest risk ADRs and emphasis should be given for female and clinically advanced patients.

## Introduction

According to The Joint United Nations Program on HIV and AIDS (UNAIDS), through 2018, 74.9 million people worldwide have been infected with HIV. Since the beginning of Human Immunodeficiency Virus (HIV) epidemic, around 74.9 million people have become infected and about 32 million people have died from the Acquired Immunodeficiency syndrome (AIDS) related illnesses. Globally, there were 37.9 million people living with HIV and 62% of people are on Antiretroviral Therapy (ART) in 2018 [1]. By 2015 Sub-Saharan Africa (SSA) shared three forth of the total HIV-infected people and almost the same amount of AIDS related deaths [2]. In Ethiopia by 2018, there were an estimated 610,335 people living with HIV and by the end of 2017, 415, 578 adults and 21,385 children under the age of 15 were on ART [3,4].

Highly active anti-retroviral therapy (HAART) has an improving effect on the quality of life and also on the course and treatment of HIV-infected patients [5]. In the past decades AIDS-related illness is declined mainly due to the continuous scale-up of HAART [6]. From 2010 up to 2018, new HIV infections and the annual number of global deaths from AIDS-related illness have been declined by 16% and 33% respectively [1]. Previously, Ethiopia was also one of the countries with the higher number of AIDS related death, but recent reports shows that there is a remarkable reduction from 83,000 deaths in 2000 to 15,600 in 2017 because of HAART implementation [7].

Despite its high potential for the management HIV infection, HAART may result mild to serious adverse drug reactions [8,9]. Adverse drug reactions (ADRs) are responding to a medicine which is noxious and unintended, and which occurs at doses normally used in man for the prophylaxis, diagnosis, or therapy of disease, or for the modification of physiological function [10].

ADRs negatively affect both the health care system and patients’ quality of life. Reports from some countries indicate that adverse drug reactions are the 4^th^ to 6^th^ largest cause for mortality and 10–20% of hospitalization. Due to drug related problems, some countries also spend up to15-20% of their healthcare budget [11,12].

In Africa a study done in Nigeria by 2015 on the safety of the drugs majority proportion of ADRs was accounted by antiretroviral drugs [13]. Similarly, in Ethiopia, the pharmacovigilance center report from 2009–2011 indicates that more than three forth of the adverse drug events are attributed to antiretroviral drugs followed by the anti-tubercular drugs [14]. On a study done in Jimma specialized hospital more than half (65.5%) of HIV patients evidenced to develop at least one adverse drug reaction to antiretroviral drugs [15].

The ADRs are known to result in loss of patient’s confidence on the safety of medicines and the national ART program which lead to poor adherence or total discontinuation of taking these life-prolonging medicines. Poor adherence is, in turn, known to lead to treatment failure and the development of resistance of the virus leading to reduced efficacy [9,16,17]. Moreover, ADRs secondary to ART are one of the main causes of, treatment discontinuation, changes in ART regimens, hospitalization, prolonged hospital stays and high costs and even death [18–23].

Despite, the current Sustainable development goal (SDG) target of ending communicable disease, including AIDs through increasing the uptake of ART [24], globally, toxicities result in about one out of four HIV infected patients to discontinue and not adhere to their regimen [8]. Likewise, in South Africa, 40% of the patient had a first line anti-retroviral discontinuation by three years of ART due to ADRs [18]. In Ethiopia, studies showed that toxicity of the drugs is the main causes (58%) for the change of the first-line regimen among HIV patients on antiretroviral therapy, which affects the limited number of our treatment options. It is also one of the primary predictor of poor adherence [25–27]. A study conducted in south-west Ethiopia on mortality from adverse drug reaction-related hospitalizations show that 1.5% death. Drug-induced hepatotoxicity and acute kidney injury which is mostly attributed by anti-retroviral drugs (Efavirenz (EFV) and Tenofovir(TDF) following anti-tuberculosis drugs (Isoniazid and Pyrazinamide) are known to be the major cause of death [20].

Provided negative effect of ADRs, Ethiopia has been implementing several activities at the national level through Pharmacovigilance center. Accordingly, the country has sent letters to health care providers about the safety concern describing how it may affect present patients on the medicine and future prescribing [28]. At the facility level, as some studies indicate the commonly used solution to avert such problem with the health care provider is reassurance, single drug change and regimen modification [29]. A study done at Gondar identified that positive emotional, social support seeking, taking other medications, information seeking and non-adherence as a mechanism for coping ART related ADRs by the patients [27].

Studies from different part of the world indicates sex, age, educational status, occupation, base line WHO clinical stage, baseline CD4 count, opportunistic infection prophylaxis, anti-tuberculosis treatments, duration of use of ART and regimen type have an effect on the development of ADRs among adult HIV patient on ART [29–35]. Factors such as nutritional status, Co-morbid non AIDS related chronic disease and substance use was not well studied and would be assessed in this study. This study aimed to determine the incidence of ADR among patients on HAART and its predictors.

## Methods

### Study setting and design

This study was conducted in Arba Minch town, public health facilities which give ART services, Arba Minch General Hospital, Arba Minch and Secha Health Center. Arba Minch town is the administrative center of Gamo zone. It is located 505 km southwest from Addis Ababa, the capital city of Ethiopia and 275 km southwest of Hawassa, capital city of SNNPR.

Arba Minch General Hospital is now providing health services for about 2,007,143 peoples which came from the catchment area. ART service was started on 2004, and from the time the hospital starts to give HIV care service about 4,000 HIV patients were enrolled for ART. Among them 3,159 were initiated ART. Currently, 1,751 HIV patients were on treatment at the ART clinic, of which 1,628 were adult. The remaining patients were transferred out to the nearby health facilities. Arba Minch health center start to enroll HIV patients on 2005 but it starts to initiate ART on 2007. Starting from this time, 619 patients were initiated ART and, currently 400 patients are on treatment and of which 374 are adult. An institution-based retrospective cohort study was conducted by reviewing medical records of HIV patients who initiated ART between January 1, 2013 and December 30, 2018. The medical records review was done from April 20 up to May 20, 2020.

### Population of the study

All charts of adult HIV patients who initiated ART at Arba Minch Town public health facilities were considered as source population, and all adult HIV patients initiated ART between January 1, 2013 and December 30, 2018 in Arba Minch Town public health facilities were considered as the study population.

### Eligibility criteria

Adult HIV patients who initiated ART between January 1, 2013 and December 30, 2018in Arba Minch town public health facilities were included, whereas HIV patients on ART whose medical record were incomplete (those records lacking information on date of ART initiation, the outcome of interest or those records missing more than 20% independent variables) were excluded from this study.

### Sample size determination

In this study sample size was determined by using single population proportion formula by taking the proportion 22.1%from previous study, which was done on adult HIV patients in Ethiopia [33]. With the following assumptions under consideration; desired degree of precision of 4%, and 95% of confidence interval it provides 414 and with the consideration of 10% withdrawal it gives 456.

### Sampling technique

This study was carried out in Arba Minch Town public health facilities that provide ART service during the specified study period, Arba Minch General Hospital and Arba Minch Health Center. Therefore, the total sample size was proportionately allocated for this two selected health facilities based on the number of study population. The proportional allocation was as follows: 303 out of 614 from Arba Minch general hospital and 153 out of 310 from Sikela health center. A total of 968 medical cards were reviewed, of which 20 and 24 were excluded due to ineligibility and incompleteness (more than 20% of variables were incomplete) respectively. Thus, medical records of HIV patients who fulfilled the inclusion criteria were included with the above-mentioned public health facilities until the calculated sample size was attained. Simple random sampling technique with computer generated random number was used and medical records of those HIV patients who start ART between January 1, 2013 and December 30, 2018 were considered as a sample frame.

### Data collection methods and procedure

The data extraction checklist was prepared in the English language after looking routine standard ART monitoring charts, HIV patient’s medical records, ART registers and relevant peer reviewed similar studies. To gather all the available relevant data necessary for conducting this study, ART registers and ART patient’s individual medical folder were reviewed. The checklist had consisted socio-demographic, clinical, laboratory, medical, and behavioral characteristics related questions. Data were collected by six diploma nurse professionals and two supervisors who had BSc nurses. Two days training was given by principal investigators for both data collectors and supervisor on briefing the general objective of the study, and discussing the contents of the checklist and how it is to be filled. Data collectors and supervisors were applied all necessary protective measures of Corona virus disease 2019(COVID-19); such as face mask and alcohol based rub during medical record review time, and also they maintained physical distancing safeguard themselves as well as field workers and others.

### Data quality assurance

To assure the data quality, data collection tool was prepared after reviewing of HIV patients’ medical records, ART registers, and relevant peer reviewed similar studies. The collected data was delivered to the supervisor every day and the principal investigator every other day in order to take immediate action when inconsistencies and problems happen on the recorded data. The completeness of the data was checked by data collectors during data collection and after data collection by the supervisor and principal investigator (PI). The overall activity of data collection was also supervised and coordinated by the PI. Finally, data completeness is reassured and data cleaning was made by the principal investigator.

### Data processing and analysis

Data were coded, cleaned, and entered using Epi-data software version 4.4.2, exported to STATA version 14 (STATA Corporation, College Station Texas) software for analysis. The presence of missing values, possible outliers, and multi-collinearity were checked through exploratory analysis. Descriptive analyses, including median and inter-quartile range (IQR) for numerical variables, frequencies and proportions for categorical variables was performed to characterize the study participants based on the studied variables and for the type of adverse reactions also. Deletion (variable dropping and complete case analysis) strategy was used to handle Missed values.

Person-time incidences were estimated. The incidence rate of ADR were expressed as the number of patients with at least one occurrence of the given event per 100 person-years, the numerator was defined as the number of patients who have at least one adverse reaction recorded in the medical charts, whereas the denominator was defined as the sum of time contributed by each individual corresponding to the interval between the date of the first ART initiation up to the date of the first registered adverse reaction. For those without any registered adverse reactions, the interval between the date of the first day of ART initiation and the date of the last medical visit was considered. Kaplan-Meier survival curve with the log-rank test was fitted to check the presence of a difference in experience of ADR among the categorical variables.

Since the time of exposure, i.e., the first day at which the patient starts to take ART, to outcome, i.e., adverse reactions, was known and heterogeneous for each individual under investigation they were suitable for survival analysis. To show an association between the covariates and timing of ADRs and differences in survival rate Kaplan Meier curve were used and log-rank test was used to test the equality of the curves between categories of covariates on the timing to first ADR events. To estimate and interpret survivor functions from survival data overall Kaplan-Meier survivor function curve was used.

The magnitude of the association between predictive factors and adverse reactions was estimated in terms of the hazard ratio (HR) at 95% confidence interval by using Cox proportional hazards model. The independent effect of selected variables on the occurrence of at least one adverse reaction was determined by multivariable analysis. In the bi-variable analysis, the variables with p-value < 0.25 were eligible for multivariable analysis. Finally, variables with p-values less than 0.05 in the multivariable Cox regression were considered as independent predictors.

The likelihood ratio test was used to compare models and the proportional hazard assumption was assessed by using these three approaches; graphical, goodness of fit test and time dependent variables.

### Measurements of the variables

Adverse drug reaction:

Event: occurrence of ADRs after initiation of ART.

In this study an adverse drug reaction to ART was defined as the occurrence of at least one of the following undesirable effect registered in the patient’s follow-up card (Nausea, Diarrhea, Fatigue, Headache, Numbness/tingling/pain, Skin Rash, Anemia, Abdominal pain, Jaundice, Fat change, Dizzy, anxiety, nightmare, depression) and for this study only the first event which occurred following the date of the first prescription of anti-retroviral drugs was under investigation.

Censored: in this study censors were individuals like lost to follow up, dead, transferred out, and the individual who does not experience the event at the end of the follow up period.

ART drug adherence: adherence is the extent to which a client’s behavior coincides with the prescribed regimen as agreed upon through a shared decision making process between the client and the health care provider. And it was described as good adherence (≥ 95% adherence that is, missing only ≤ 2 out of 30 doses or missing ≤ 3 from the 60 doses), fair adherence (85–94% adherence that is, missing 3 to 5 doses out of 30 tabs or 3 to 9 tablets from 60 doses) and poor adherent (less than 85% adherence that is, missing ≥ 6 tablets out of 30 tabs or > 9 tabs from 60 tabs) [4].

Functional status of patient at initiation of ART: this is to assess the physical, functional status of the patients at initiation of ART and it was described as working (able to perform usual work inside or outside the home), ambulatory (able to perform activity of daily living, not able to work) and bed ridden (not able to perform activities of daily living) [4].

WHO clinical stage at initiation of ART: it is a classification of HIV patients at initiation of ART treatment based on clinical illnesses as defined by WHO clinical staging of HIV patient and in this study it was categorized as non-advanced HIV disease (WHO stage I and II) and advanced HIV disease (WHO stage III and IV) [36].

### Ethical consideration

Ethical approval was obtained from Institutional Research Ethics Review Board (IRB) of Arba Minch University, College of Medicine and Health Science. Following the approval, support letter to the concerned bodies was given from Post Graduate Coordination Office of Arba Minch University. Permission was obtained from Arba Minch town Health Office, Arba Minch General Hospital and Health Center. Clear information about the concept, purpose, procedure, requirements, significance of this study and investigator were given for the selected study facility administrators, for those supportive staff expected to involve and those health professionals that works in the ART clinic. The confidentiality of the information obtained throughout the study was handled carefully and kept locked. Then, informed, voluntary, written and signed consent was also obtained from the head of the Hospital in accordance with Declaration of Helsinki. Moreover, the IHRERC of the Arba Minch University has not required the patient parental consent to review their medical records since it is retrospective nature of the study. Information obtained from patient medical record was kept confidential and used only for this particular study.

## Results

### Baseline characteristics of the study participants

#### Socio-demographic characteristics of the respondents

A total of 456 HIV positive adults’ medical records 303(66.4%) from Arba Minch general hospital and 153 (33.6%) from Arba Minch health center was reviewed. The median and IQR of the age of the clients at the initiation of ART was 30 and 10 years, respectively, and of which nearly half of (46.27%) the participants were in the age group between 25 and 34 years. Approximately two third of the participants (61.18%) were females. Regarding the level of education, 111 (24.34%) had no formal education and 52 (11.4%) were tertiary educated. Regarding the residence, 337 (82.68%) was from urban area and 79 (17.32%) rural area and among all clients 82% resided in the facilities catchment area and the rest were outside the facilities catchment area (Table 1).

**Table 1:** Socio-demographic characteristics of HIV positive adults at initiation of HAART in Arba Minch town public health facilities from January 2013 to December 2018 (N = 456).

Variables	Category	Frequency	Percentage
Health facility	Hospital	303	66.4
	Health center	153	33.6
Sex	Male	177	38.82
	Female	279	61.18
Age at initiation ART	15–25	73	16.01
	25–34	211	46.27
	35–44	124	27.19
	≥45	48	10.53
Marital status	Single	85	18.64
	Married	253	55.48
	Separated or divorced	87	19.08
	Widowed	31	6.80
Educational status	No formal education	111	24.34
	Primary	154	33.77
	Secondary	139	30.48
	Tertiary	52	11.4
Occupation	Governmental employee	77	16.89
	Farmer	61	13.38
	Merchant	56	12.28
	Daily laborer	153	33.55
	House wife	61	13.38
	Student	18	3.95
	Others	30	6.58
Residence	Urban	337	82.68
	Rural	79	17.32
Catchment area	Reside in facilities catchment area	374	82.02
	Not reside in facilities catchment area	82	17.98
Religion	Orthodox	309	67.76
	Protestant	120	26.32
	Muslim	25	5.48
	Others	2	0.44

### Behavioral characteristic of the respondents

Of the total reviewed patients medical records, 86.4% showed good adherence to ART and more than ninety percent of patients were disclosed their HIV status. Regarding the substance use, more than one quarter of the patients (27.41%) had experience of alcohol user, and only 1.97% of had smoking experience (Table 2).

**Table 2:** Behavioral characteristics of HIV positive adults on HAART at Arba Minch town public health facilities, from January 2013 to December 2018 (N = 456).

Variables	Categories	Frequency	Percentage
**Adherence**	Good	394	86.4
	Fair	19	4.17
	Poor	43	9.43
**Disclosure status**	Yes	423	92.76
	No	33	7.24
**Alcohol use**	Yes	125	27.41
	No	331	72.59
**Tobacco use**	Yes	9	1.97
	No	447	98.03

### Clinical and immunological characteristics

More than three forth (77.41%) of patients were on WHO category, stage I and II at the time of HAART initiation. Out of the total subjects, 52 patients lack CD4 information on the base line and the median and IQR of CD4 count for the patients was 347 and 236 respectively. The mean and standard deviation of the BMI of the patients were 21.18 ±2.88 and based on their BMI 15.13% and 1.54% of participants were moderately and severely malnourished respectively. On the other hand, 33.33% were also infected with opportunistic infections and 3.07% had other non AIDS related disease (Table 3).

**Table 3:** Clinical and immunological characteristics of HIV positive adults at initiation of HAART at Arba Minch town public health facilities, January 2013 to December 2018 (N = 456).

Variables	Category	Frequency	Percentage
Baseline WHO clinical stage	Stage 1	172	37.72
	Stage 2	181	39.69
	Stage 3	73	16.01
	Stage 4	30	6.58
CD4 count at initiation of ART	≤ 200 cells/μl	74	16.2
	>200 cells/μl	330	72.4
	Missed	52	11.4
Functional status at initiation of ART	Working	402	88.15
	Ambulatory	47	10.31
	Bedridden	7	1.53
BMI	>18.5	380	83.33
	16–18.5	69	15.13
	<16	7	1.54
OIs at initiation of ART	Yes	152	33.33
	No	304	66.67
Other non AIDS related comorbidities	Yes	14	3.07
	No	442	96.93

### ART and other medication related characteristics

In this study, more than three forth (80.92%) of prescribed HAART regimen in initiation for the patients were a combination TDF-3TC-EFV. More than half of (53.39%) patients had received Cotrimoxazole prophylaxis and 91.67% of patients had received Isoniazid prophylaxis. Among all, 36(8.11%) of patients had a history of anti TB medications (Table 4).

**Table 4:** ART and other medication related characteristics of HIV positive adults at initiation of HAART at Arba Minch town public health facilities, January 2013 to December 2018 (N = 456).

Variables	Category	Frequency	Percentage
ART regimen	1c, AZT-3TC-NVP	40	8.77
	1d, AZT-3TC-EFV	12	2.63
	1e, TDF-3TC-EFV	369	80.92
	1f, TDF-3TC-NVP	35	7.68
CPT	Yes	243	53.39
	No	213	46.71
IPT	Yes	418	91.67
	No	38	8.33
Anti-TB treatment	Yes	36	7.89
	No	420	92.11

### Incidence of adverse drug reaction

During the six year retrospective follow up, 79 (17.32%) had developed ADRs, 6 (1.3%) died, 74 (16.2%) were transferred out to other health facility, 36(7.9%) were lost to follow up, and 261 (57.2%) were on follow up without experiencing ADRs. Patients were followed for a minimum of 1month and maximum of 87 months with a total of 14,903 person months of observation and the median observation time was 27 months with IQR 35 months. Overall, there were 79 incidents of ADR reported during 14,903 person-months of observation, or 1,241.9 person-years. And the incidence density rate was 0.53/100 person’s month (CI: 0.42–0.66/100 person’s month), or 6.36 per 100 Pearson years of observation, (7.52/100PY among females and 4.54/100PY among males). Of these, more than half (53.16%) of ADRs were occurred in the first one year of observation and over three forth (78.5%) of ADRs occurred within two years of follow up. Survival probability at the 6^th^, 12^th^, 24^th^ and by the end of the study was 0.95, 0.84, 0.82 and 0.69 respectively (Table 5).

**Table 5:** Life table for adverse drug reaction survival among adult HIV positive patients in Arba Minch town public health facilities, southern Ethiopia 2020.

Interval in months	Number Entering Interval	Number Withdrawing during Interval	Number Exposed to Risk	Number of Terminal Events	Proportion Terminating	Probability of Surviving	Cumulative Probability Surviving at end of Interval	95% CI
0–12	456	42	435	37	0.09	0.91	0.91	0.88,0.93
12–24	377	98	328	26	0.08	0.92	0.84	0.80,0.87
24–36	253	65	220.5	8	0.04	0.96	0.81	0.76,0.84
36–48	180	55	152.5	2	0.01	0.99	0.80	0.75,0.83
48–60	123	57	94.5	1	0.01	0.99	0.79	0.74,0.83
60–72	65	29	50.5	4	0.08	0.92	0.73	0.64,0.79
72–84	32	25	19.5	1	0.05	0.95	0.69	0.58,0.77
84–96	6	6	3	0	0.00	1.00	0.69	0.58,0.77

**Fig 1:**
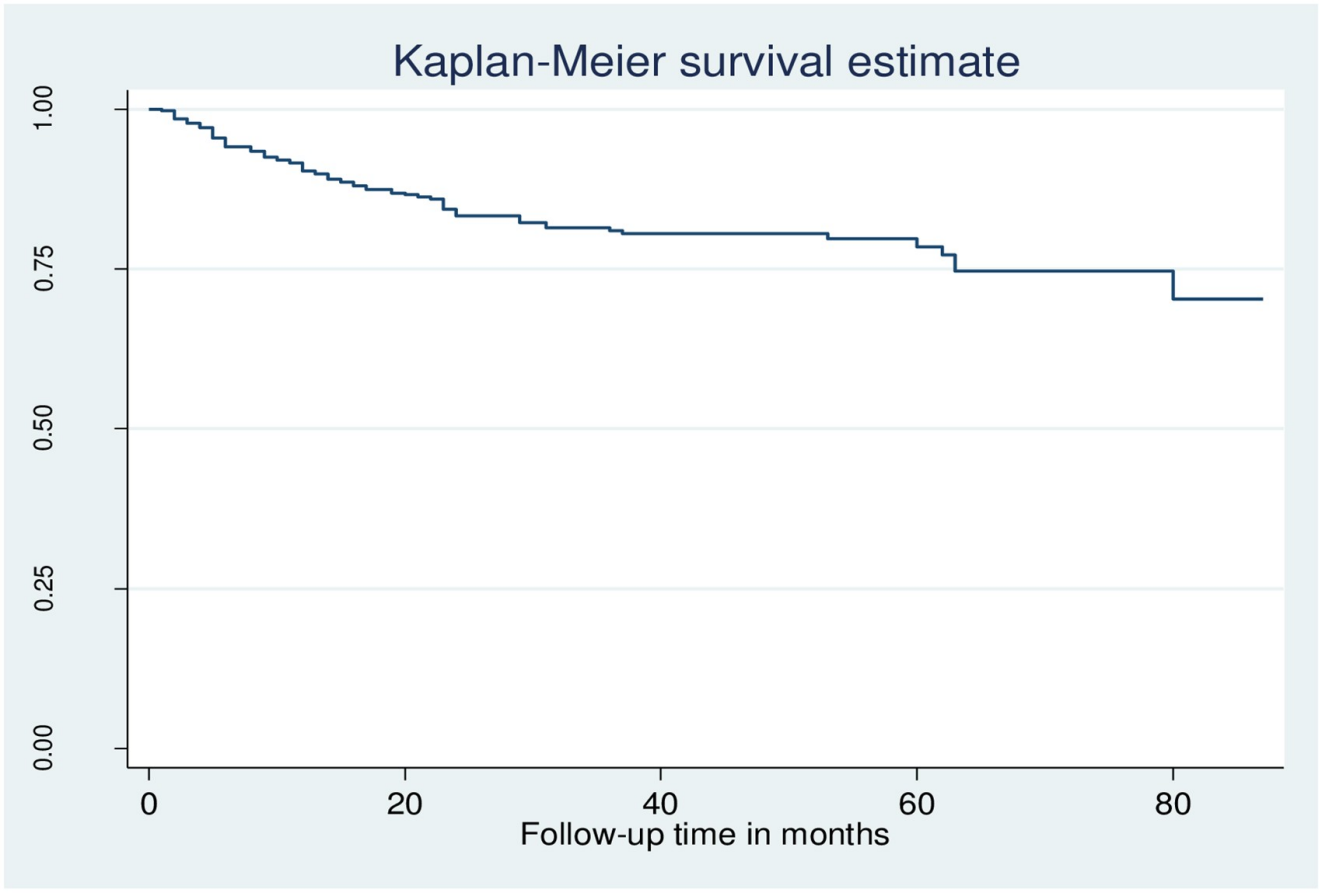
The overall Kaplan-Meier survival estimate curve of adult HIV positive patients on ART in public health facilities of Arba Minch town, southern Ethiopia, 2020.

The overall Kaplan-Meier survival estimate curve showed that incident of ADR was highest in the first two years of ART initiation and reduced over the time of ART (Fig 1).

**Fig 2:**
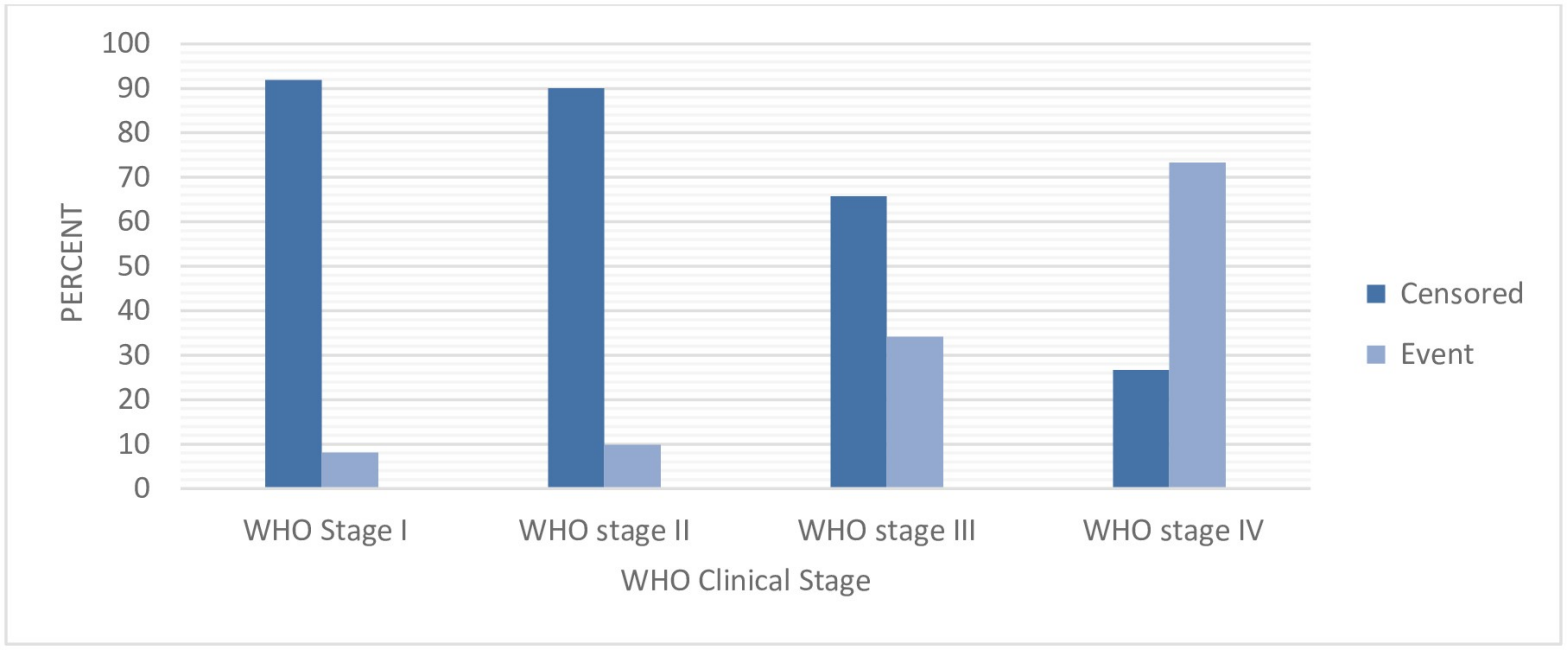
Distribution of ADRs by baseline WHO clinical stage among adult HIV patients on ART in public health facilities of Arba Minch town, southern Ethiopia, 2020.

The incidence of ADRs was higher among those patients who start ART at advanced clinical stage compared to non-advanced once (Fig 2).

**Fig 3:**
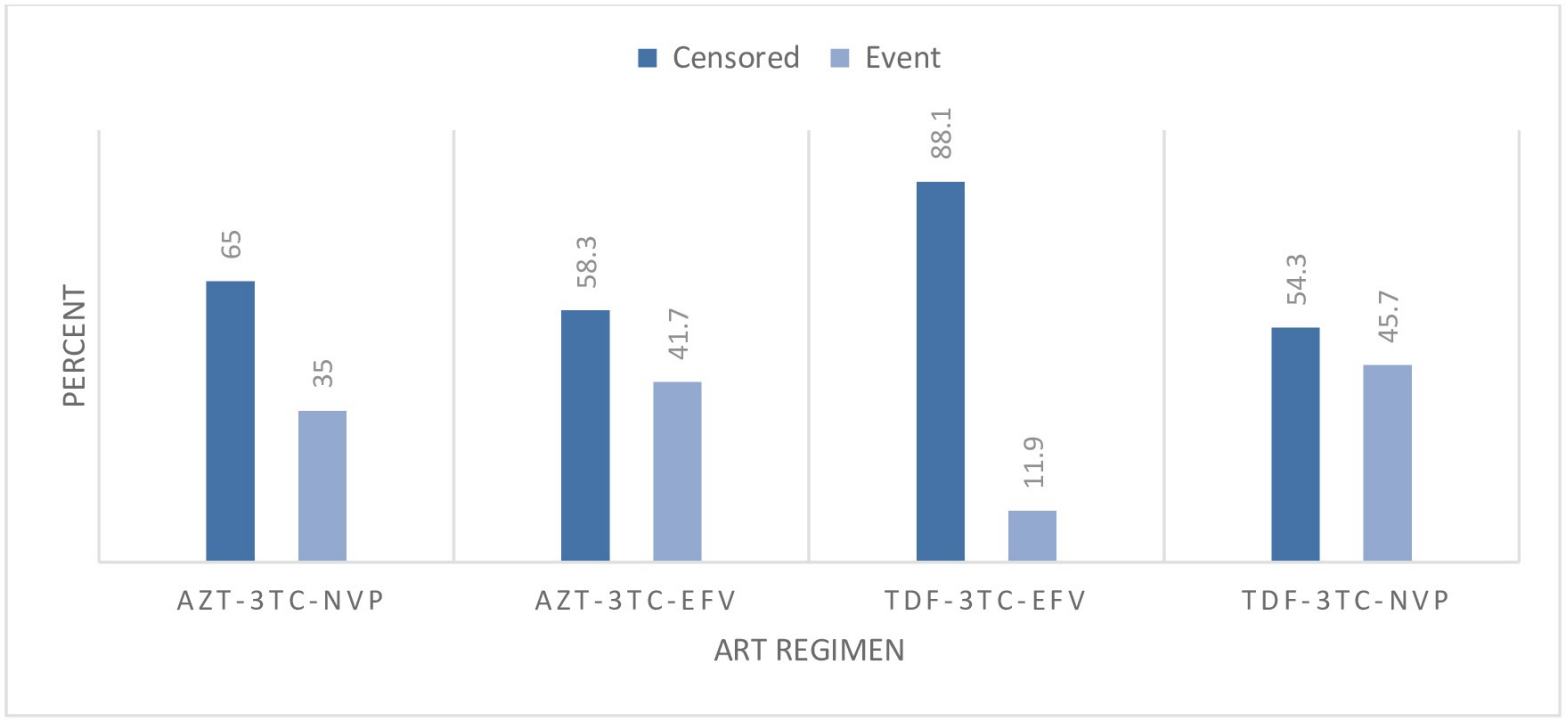
Distribution of ADRs by initial ART regimen type among adult HIV positive patients on ART in public health facilities of Arba Minch town, southern Ethiopia, 2020.

The incidence of ADR was lower among those patients who were on TDF-3TC-EFV as compared to other regimens (Fig 3).

To test equality of survival curves of different categorical explanatory variables, Cochran-Mantel Haenszel Log rank test was performed. The test statistics showed that there was a significant difference in survival function for different categorical variables (Table 6).

**Table 6:** Log-rank test for equality of survivor functions between categories of covariates.

Variables	Chi 2 (X2)	Pr>chi2
Sex	4.56	0.028
Occupation	17.43	0.0148
Disclosure	4.39	0.036
Functional status	26.49	<0.001
BMI	6.60	0.0368
WHO clinical stage	79.01	<0.001
CD4 count	25.54	<0.001
Opportunistic infection	9.33	0.0023
Regimen type	45.99	<0.001
Anti-tuberculosis	41.21	<0.001
CPT	32.9	<0.001
IPT	32.47	<0.001
Type of health facility	16.45	<0.001

**Fig 4:**
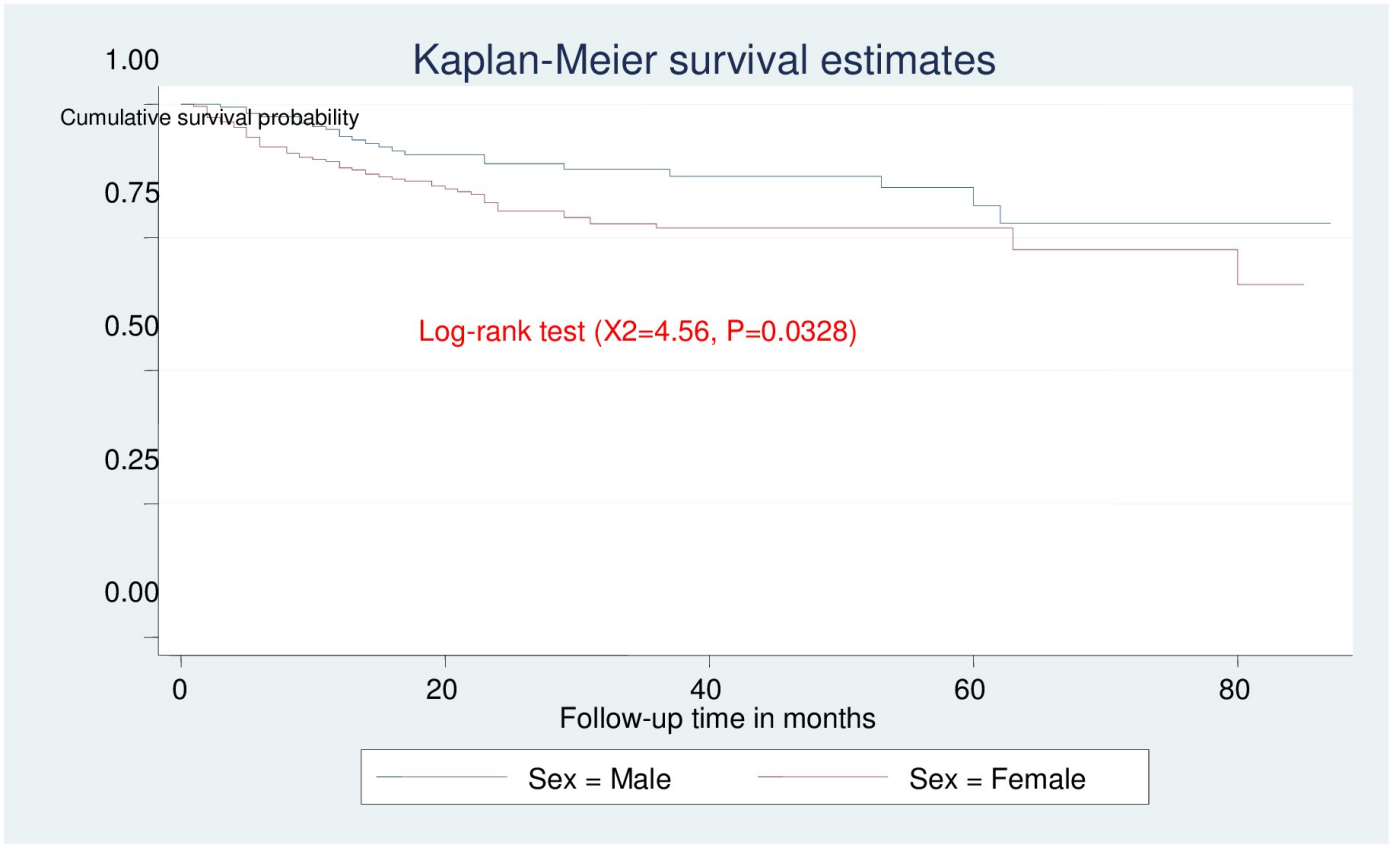
Kaplan-Meier survival estimate for sex among adult HIV patients on ART in public health facilities of Arba Minch town, southern Ethiopia, 2020.

**Fig 5:**
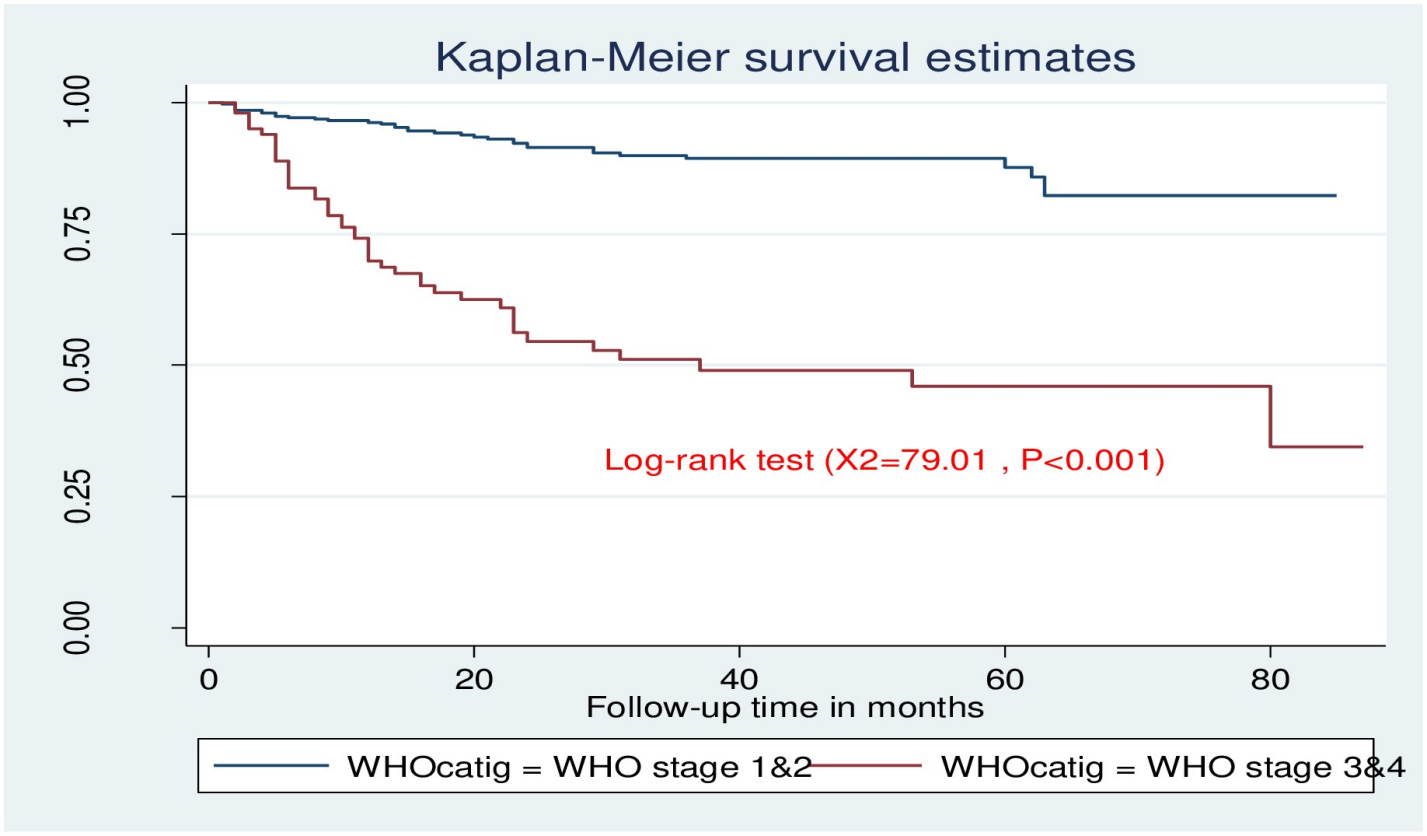
Kaplan-Meier survival estimate for base line WHO clinical stage among adult HIV patients in public health facilities of Arba Minch town, southern Ethiopia, 2020.

In this study, there was significance differences survival time among female and male (Fig 4). Those study participants who are at advanced clinical stage had lower survival time as compared to those who are non-advanced clinical stage, and the survival time difference between the groups was found statistically significant (Fig 5).

### Predictors of adverse drug reaction

On the bi-variable Cox regression, covariates such as types of health facility, sex, age category, educational status, occupation, disclosure status, functional status, WHO clinical stage at ART initiation, BMI at ART initiation, opportunistic infections, CD4 count at initiation of ART, types of regimens, experience of anti-tuberculosis treatment, cotrimoxazole preventive therapy, and isoniazid preventive therapy were found significant predictors for the occurrence of ADRs at p-value <0.2 and they were candidates for multivariable regression (Table 7).

**Table 7:** Bi-variable Cox regression for predictors of ADRs among HIV positive adults on ART at Arba Minch town public health facilities, January 2013 to December 2018.

Variables	Category	Survival status		CHR(95% CI)	P value
		Event	Censored		
Sex	Male	22(12.4)	155(87.6)	1	
	Female	57(20.4)	222(79.6)	1.69(1.03, 2.77)	0.036*
Age	15–25	5(6.8)	68(93.2)	1	
	25–34	40(18.9)	171(81.1)	2.51(0.99, 6.37)	0.052*
	35–44	23(18.5)	101(81.5)	2.66(1.01, 7.01)	0.047*
	≥45	11(22.9)	37(77.1)	2.86(0.99, 8.26)	0.051*
Marital status	Single	14(16.5)	71(83.5)	1	
	Married	41(16.2)	212(83.8)	0.91(0.49, 1.68)	0.778
	Separated or divorced	18(20.7)	69(79.3)	1.11(0.55, 2.24)	0.763
	Widowed	6(19.3)	25(80.7)	1.16(0.45, 3.03)	0.753
Education	No formal education	13(11.7)	98(88.3)	1	
	Primary	30(19.5)	124(80.5)	1.99(1.04,3.83)	0.037*
	Secondary	28(20.1)	111(79.9)	1.92(0.99,3.72)	0.051*
	Tertiary	8(15.4)	44(84.6)	1.57(0.64,3.79)	0.318
Occupation	Governmental employee	15(19.5)	62(80.5)	1	
	Farmer	19(31.1)	42(68.9)	2.03(1.03, 4.00)	0.041*
	Merchant	8(14.3)	48(85.7)	0.68(0.29,1.62)	0.394
	Daily laborer	21(13.7)	132(86.3)	0.66(0.34,1.29)	0.226
	House wife	10(16.4)	51(83.6)	0.89(0.40,1.99)	0.781
	Student	2(11.1)	16(88.9)	0.96(0.22,4.25)	0.965
	Others	4(13.3)	26(86.7)	0.72(0.24,2.19)	0.573
Residence	Urban	67(17.7)	310(82.3)	1	
	Rural	12(15.2)	67(84.8)	0.97(0.52, 1.79)	0.918
Catchment area	Reside in facilities catchment area	66(17.6)	308(82.4)	1	
	Not reside in facilities catchment area	13(15.8)	69(84.2)	1.06(0.55, 1.82)	0.986
Adherence	Good	73(18.5)	321(81.5)	1	
	Fair	2(10.5)	17(89.5)	0.65(0.23, 1.78)	0.400
	Poor	4(9.3)	39(90.7)	0.55(0.13, 2.23)	0.402
Disclosure	Disclosed	78(18.4)	345(81.6)	1	
	Not disclosed	1(3)	32(97)	0.16(0.02, 1.15)	0.068*
Alcohol use	Yes	25(20)	100(80)	1.20(0.74, 1.93)	0.452
	No	54(16.3)	277(83.7)	1	
Functional status	Working	59(14.6)	344(85.4)	1	
	Ambulatory	17(36.2)	30(63.8)	3.04(1.77, 5.22)	<0.001*
	Bedridden	3(42.8)	4(57.2)	5.75(1.80,18.43)	0.003*
WHO clinical stage	WHO stage I and II	34(9.6)	321(90.4)		
	WHO stage III and IV	45(44.5)	56(55.5)	5.94(3.80, 9.30)	<0.001*
BMI	>18.5	59(15.5)	321(84.5)	1	
	16–18.5	19(27.5)	50(72.5)	1.94(1.15, 3.25)	0.012*
	<16	1(14.3)	6(85.7)	1.12(0.15, 8.10)	0.909
OIs	Yes	38(25.0)	114(75.0)	1.96(1.26, 3.06)	0.003*
	No	41(13.5)	263(86.5)	1	
Other non AIDS related co morbidities	Yes	3(21.4)	11(78.6)	1.58(0.49,5.01)	0.439
	No	76(17.2)	366(82.8)	1	
CD4 count	≤ 200 cells/μl	25(33.7)	49(66.3)	3.38(2.04, 5.59)	<0.001*
	> 200 cells/μl	39(11.8)	291(88.2)	1	
ART regimen	1e, TDF-3TC-EFV	44(11.9)	325(88.1)	1	
	1d, AZT-3TC-EFV	5(41.7)	7(58.3)	3.27(1.28, 8.36)	0.013*
	1c, AZT-3TC-NVP	14(35.0)	26(65.0)	3.50(1.91, 6.43)	<0.001*
	1f, TDF-3TC-NEV	16(45.7)	19(54.3)	4.93(2.77, 8.76)	<0.001*
Anti-TB treatment	Yes	19(52.8)	17(47.2)	4.67(2.77, 7.86)	<0.001*
	No	60(14.3)	360(85.7)	1	
CPT	Yes	66(27.2)	177(72.8)	4.81(2.65, 8.72)	<0.001*
	No	13(6.1)	200(93.9)	1	
IPT	Yes	61(14.6)	357(85.4)	0.24(0.14, 0.41)	<0.001*
	No	18(47.4)	20(52.6)	1	
Health facility	Hospital	66(21.8)	237(78.2)	1	
	Health center	13(8.3)	140(91.7)	0.31(0.17, 0.56)	<0.001*

Note:

* indicates p-value less than 0.25, 1 indicates reference value.

On the other hand, marital status, residence, catchment area, religion, adherence, alcohol and tobacco use, other non AIDS related co-morbidities were not found to be predictors of ADRs as a result they were not fitted to multivariate analysis.

During multiple variable Cox regression, sex, WHO clinical stage, regimen type and type of health facility were identified as an independent predictor of ART related ADRs. At any given time, the risk of developing ADRs among females was 2.35 times that of males [ADR: 2.35; CI (1.18, 4.69)]. Regarding WHO clinical stage, compared to non-advanced clinical stages (WHO stage I and II) at any given time, the risk of ADRs was 3 times higher among advanced clinical stages (WHO stage III and IV). The risk of experiencing an ADR was at any given time higher among AZT and NVP containing regimens compared to TDF and EFV containing regimens. Moreover, the risk of ADR reduced by 59% among those HIV patients who were on ART at the health center compared to those from the hospital (Table 8).

**Table 8:** Multiple-variable Cox regression for predictors of ADRs among HIV positive adults on ART at Arba Minch town public health facilities, January 2013 to December 2018.

Variables	Category	Survival status		CHR(95%CI)	AHR (95% CI)	P-value
		Event	Censored			
Sex	Male	22(12.4)	155(87.6)	1	1	
	Female	57(20.4)	222(79.6)	1.69(1.03,2.77)*	2.35(1.18, 4.69)	0.015**
Base line age	15–24	5(6.8)	68(93.2)	1	1	
	25–34	40(18.9)	171(81.1)	2.51(0.99,6.37)*	2.85(0.82, 9.84)	0.098
	35–44	23(18.5)	101(81.5)	2.66(1.01,7.01)*	3.41(0.88, 13.19)	0.075
	≥45	11(22.9)	37(77.1)	2.86(0.99,8.26)*	3.70(0.91, 15.16)	0.068
Education	No formal education	13(11.7)	98(88.3)	1	1	
	Primary	30(19.5)	124(80.5)	1.21(1.04,3.83)*	1.77(0.77, 4.10)	0.178
	Secondary	28(20.1)	111(79.9)	1.92(0.99,3.72)*	2.16(0.88, 5.31)	0.092
	Tertiary	8(15.4)	44(84.6)	1.57(0.65,3.79)	1.10(0.27, 4.29)	0.912
Occupation	Governmental employee	15(19.5)	62(80.5)	1	1	
	Farmer	19(31.1)	42(68.9)	2.03(1.03,4.00)*	1.44(0.53, 3.95)	0.472
	Merchant	8(14.3)	48(85.7)	0.68(0.29,1.62)	0.58(0.17, 1.96)	0.383
	Daily laborer	21(13.7)	132(86.3)	0.66(0.34,1.29)*	1.21(0.44, 3.39)	0.705
	House wife	10(16.4)	51(83.6)	0.89(0.40,1.99)	1.85(0.61, 5.59)	0.276
	Student	2(11.1)	16(88.9)	0.96(0.22,4.25)	2.73(0.23,32.22)	0.423
	Others	4(13.3)	26(86.7)	0.72(0.24,2.19)	1.93(0.51, 7.36)	0.332
Disclosure	Disclosed	78(18.4)	345(81.6)	1	1	
	Not disclosed	1(3)	32(97)	0.16(0.02,1.15)*	0.88(0.11, 7.20)	0.912
Base line Functional status	Working	58(14.6)	344(85.4)	1	1	
	Ambulatory	17(36.2)	30(63.8)	3.09(1.80,5,32)*	1.96(0.83, 4.59)	0.121
	Bedridden	3(42.8)	4(57.2)	7.32(2.64,20.25)	1.93(0.35, 10.58)	0.449
Base line WHO clinical stage	Stage I and II	34(9.6)	321(90.4)	1	1	
	Stage III and IV	45(44.5)	56(55.5)	5.94(3.80,9.30)*	3.00(1.22, 7.37)	0.017**
Variables	Category					
Base line BMI	>18.5	59(15.5)	321(84.5)	1	1	
	16–18.5	19(27.5)	50(72.5)	1.94 (1.15,3.25)*	0.61(0.26, 1.41)	0.244
	<16	1(14.3)	6(85.7)	1.12 (0.15, 8.10)	0.34(0.04, 2.21)	0.349
OIs	Yes	38(25.0)	114(75.0)	1.96 (1.26,3.06)*	0.94(0.46, 1.95)	0879
	No	41(13.5)	263(86.5)	1	1	
Base line CD4 count	≤200 cells/μl	25(33.7)	49(66.3)	0.29(0.18, 0.49)*	1.53(0.77, 3.14)	0.214
	>200 cells/μl	39(11.8)	291(88.2)	1	1	
ART regimen	TDF-3TC-EFV	44(11.9)	325(88.1)	1	1	
	AZT-3TC-EFV	5(41.7)	7(58.3)	3.27(1.28, 8.36)*	1.88(0.41, 8.56)	0.410
	AZT-3TC-NVP	14(35.0)	26(65.0)	3.50(1.91, 6.43)*	2.84(1.26, 6.41)	0.012**
	TDF-3TC-NVP	16(45.7)	19(54.3)	4.93(2.77, 8.76)*	3.90(1.80, 8.45)	0.001**
Anti-TB treatment	Yes	19(52.8)	17(47.2)	4.67(2.77, 7.86)*	0.64(0.17, 2.40)	0.514
	No	60(14.3)	360(85.7)	1	1	
CPT	Yes	66(27.2)	177(72.8)	4.81(2.65, 8.72)*	2.18(0.97, 4.91)	0.059
	No	13(6.1)	200(93.9)	1	1	
IPT	Yes	61(14.6)	357(85.4)	0.24 (0.14,0.41)*	0.63(0.17, 2.28)	0.486
	No	18(47.4)	20(52.6)	1	1	
Health facility	Hospital	66(21.8)	237(78.2)	1	1	
	Health center	13(8.3)	140(91.7)	0.31(0.17, 0.56)*	0.41(0.17, 0.97)	0.043**

Note:

* P<0.2 which are candidate for multi-variable Cox regression model,

** indicates p-value<0.05, 1 indicates reference group, CI = confidence interval.

## Discussion

The aim of this retrospective cohort study was to determine the incidence and predictors of anti-retroviral drugs related ADRs among HIV positive adults on ART. The overall incidence density of ADRs was found 0.53/100 person’s month (CI: 0.42–0.66/100 person’s month), or 6.36 per 100 person-years of observation.

This finding was slightly higher than a study done in Nigeria (4.6/100PY) [30,32,37], and in Ethiopia, Bahir Dar (4.3/100 PY), Hossana (4.88/100PY). One possible explanation might be the difference in the duration of follow-up period, such as in this study follow-up time was six years compared to a study done in Nigeria, which was three years, might not be able to observe some ADRs that occur late after ART initiation and the other is difference on measurement of the events between studies, such as on study done at Bahir Dar and Hossana, only those ADRs that results patient hospitalization, or drug switch/discontinuation because of ADRs was considered for investigation.

On the other hand, this finding was lower than a study done in our country at seven teaching hospitals (Addis Ababa, Hawassa, Jimma, Haramaya, Mekelle, Gondar and Army forces hospital), where the incidence density was 9/100 PY [33]. It was also very much lower than a study done in India (52/100 PY) [38]. The possible reason for this variation might be due to differences in the type of regimen between studies. For example, in this study more than 80% of patients were started ART with TDF containing regimens compared to study done at tertiary hospitals in that study more than 70% patients start ART with AZT and Stavudin containing regimens which are more toxic than TDF. The other possible explanation might be differences in the characteristics of the population included in the studies. In this study, the majority of the study participants initiated ART at WHO clinical stage (I&II) relative to previous studies conducted at teaching hospitals in which 67.2% of subjects were at an advanced clinical stage at the initiation of ART. Moreover, the finding in this study was entirely based on retrospective data which might underestimate the actual risk of the ADRs compared to study done in Ethiopia at seven teaching hospitals, which uses both retrospective and prospective data, and India which is entirely a prospective study.

Concerning the time of ADRs, 26(32.9%), 20(25.3%) and 16 (20.25%) of the ADRs occurred within the first 6th, 12th and 24th months of ART initiation respectively. This shows that the risk of ADRs were reduced as duration of ART increases and it was highest in the first six months of observation and more than three-fourth of incidents were happened in the first two years of observation. This finding is supported by previous studies conducted in our country at Bahir Dar and seven teaching hospitals and it is also agreed with a study done at Nigeria and India [30,32,33,38].

In this study, being female was found as an independent predictor for anti-retroviral related ADRs. Females were more than two times more likely to experience ADRs as compared to males. This finding was consistent with other previous studies conducted in Brazil, where the risk of ADRs was 1.93 times higher among females compared to males and it is also supported by studies done in South Africa and Ghana, in these studies the risk of ADRs among male reduced by 50% compared to females [39–41]. Differences on the risk of ADRs between male and female might be because of a number of sex related physiological differences, such as having lower body weight, differences in activity of enzymes, reduced renal clearance due to their lower glomerular filtration rate and difference on absorption, distribution, metabolization and excretion of drugs at different rates compared with men. Additionally, women use more medications compared to men such as contraceptives, hormonal supplements, minerals and vitamins, especially during their reproductive life that might predispose them to more ADRs compared to men [42,43].

WHO clinical stage at the initiation of ART was also one of an independent predictor of ADRs among adult HIV patients on ART. In this study, those patients who were at an advanced clinical stage at the time of ART initiation had a significant risk of developing ADRs. This finding was in line with a study conducted in Bahir Dar at Felege Hiwot hospital in 2017, where the risk of developing ADRs was higher among WHO clinical stage II, III and IV compared to stage I [32]. This finding was also supported by a similar type of study conducted in Nigeria in 2015 in that study, the risk of ADRs was significantly higher among patients who start ART at advanced clinical stage (WHO stage III and IV) relative to those who start ART at earlier clinical stages(WHO stage I and II) [44]. The possible explanation for the higher incidence of ADRs among patients with advanced clinical disease might be due to an increased risk of opportunistic infections and associated with this there would be also use of multiple drugs. The study suggest that patients with multiple morbidity may have altered pharmacodynamics and pharmacokinetic mechanisms due to drug-disease interaction which predisposes them to ADRs and the use of multiple drugs also increases the risk of ADRs due to drug- drug interactions [42]. This finding implies that the need of strengthening the implementation of test and treat strategies in order to reduce developing advanced clinical stage of disease in turn to reduce ADRs among them. Moreover, the finding also indicated that the need of close follows up with patients with advanced stage of disease.

This study found that Zidovudine and Nevirapin based regimen was significantly more toxic than Tenofovir and Efaverinz based regimens. This finding was supported by a study done in Uganda in 2013 in which ART related ADRs was higher in Nevirapin containing regimen compared to Efaverinz and also study in Ethiopia at Hiwot Fana hospital, in which TDF based regimens was less toxic compared to AZT containing regimens [29,45].

In this study, there was a significant difference in the incidence of ADRs between the health care facilities where the patient received ART service. However, there were no studies that have seen a difference on the risk of ADRs among the types of health care facility in such a way previously. According to this study, the risk of ADR was reduced by 59% among those HIV patients who were on ART at the health center compared to those from the hospital. One possible explanation for this might be better follow-up and counseling of patients due to low patient flow in the health center compared to the hospital. The other possible reason could be differences in the clinical condition of patients attending at the hospital and the health center; clinically advanced patients are more likely to attend to hospitals than health centers. Moreover, in this study, advanced clinical stage at the initiation of ART was 25% among hospital patients as compared to 18.3% in the health center. There might be also differences in diagnostic capability of ADRs between health center and hospital due to difference in knowledge or educational level of the health care provider and difference in the availability of diagnostic equipment. This finding implies that the need of expanding health facilities which provides ART services at community level and the need of availing training on screening of ADRs to health professionals.

The major limitation of this study was since it was based on secondary data it was difficult to ascertain the outcome variable and also there might be a difference on the measurement of the outcome variable across health professionals, which might under/overestimate the problem.

Predictors like hemoglobin level, status of viral hepatitis infection and organ functional status indicating laboratory results were not included as they were not fully documented on patient cards, had a large amount of missed values, as a result they were totally excluded from the analysis. Thus, this finding should be interpreted with this limitation in mind.

## Conclusion

Incidence of adverse drug reaction is high during the early years of ART initiation and it decreases over the time of ART. The incidence rate of ADRs is reduced among patients on ART compared to previous studies. Being female, advanced WHO clinical stage, ART regimen and type of health care facility were an independent predictor for the occurrence of ADRs among HIV positive adults on ART in Arba Minch town public health facilities. The governments and concerned bodies should give attention to early identification of a patient by screening of risk groups and early initiation of ART. A special emphasis should be given to patients in the early years of ART initiation, since this is the time of highest incidence ADRs. Providing awareness on signs and symptoms of some of the self-recognizable ADRs should be considered, in order to early diagnosis and to manage any ADRs related to anti-retroviral drugs. Moreover, prospective study should be done for more precise measurement of the magnitude of the problem and to identify some other factors like use of herbal medications and other which can be measured better by prospective observation of patients.

## Supporting information

S1 Data (DTA)

## References

[1] UNAIDS, Global HIV & AIDS statistics 2019 fact sheet. 2019.

[2] Wang HWT, C A., Nguyen G, Kyu HH, Gakidou E, et.al, Estimates of global, regional, and national incidence, prevalence, and mortality of HIV,1980–2015: the Global Burden of Disease Study 2015. Lancet HIV, 2016. 3(8): p. e361–87. 10.1016/S2352-3018(16)30087-X 27470028 PMC5056319

[3] UNAIDS, Global AIDS monitoring report. 2017.

[4] FMOH, NATIONAL CONSOLIDATED GUIDELINES FOR COMPREHENSIVE HIV PREVENTION, CARE AND TREATMENT. August, 2018: Ethiopia.

[5] Jaquet A., et al., Antiretroviral treatment and quality of life in Africans living with HIV: 12-month follow-up in Burkina Faso. J Int AIDS Soc, 2013. 16: p. 18867. 10.7448/IAS.16.1.18867 24369739 PMC3871830

[6] UNAIDS, Joint United NationsProgramme on HIV/AIDS in UNAIDS Data. 2018.

[7] MOH, F., HIV Prevention in Ethiopia National Road Map 2018–2020, F.H.A.P.a.C. Office, Editor. November 2018.

[8] FMOH, *National Comprehensive HIV Prevention*, *Care and Treatment Training for Pharmacy Professionals Participant Manual*. 2018, Ministry of Health, Ethiopia.

[9] WHO, *A practical handbook on the pharmacovigilance of antiretroviral medicines*. 2013: Geneva 27, Switzerland.

[10] WHO, *Safety Monitoring of Medicinal Products*: *Guidelines for Setting up and Running a Pharmacovigilance Centre*. 2000, The Upsala Monitoring Centre: Uppsala, sweden.

[11] WHO, *AIDE MEMOIRE For a national strategy for safe drugs and their appropriate use*. Geneva 27, Switzerland.

[12] WHO, *The SAFETY of MEDICINES IN PUBLIC HEALTH PROGRAMMES*: *Pharmacovigilance an essential tool*. 2006: Geneva 27, Switzerland.

[13] Awodele O., et al., *Patterns of adverse drug reaction signals in NAFDAC pharmacovigilance activities from January to June 2015*: *safety of drug use in Nigeria*. Pharmacol Res Perspect, 2018. 6(5): p. e00427. 10.1002/prp2.427 30324768 PMC6175912

[14] EFMHACA, *Importance of Active surveillance and Cohort Event monitoring on ARV medicines in Ethiopia* in *Pharmacovigilance newsletter* 2016, pharmacovigilance center Ethiopia.

[15] Wubishet B.L., Adverse Effects and Regimen Switch among Patients on Antiretroviral Treatment in a Resource Limited Setting in Ethiopia. Pharmacovigilance, 2013. 1.

[16] Assemie M.A., et al., Treatment failure and associated factors among first line patients on highly active antiretroviral therapy in Ethiopia: a systematic review and meta-analysis. Glob Health Res Policy, 2019. 4: p. 32. 10.1186/s41256-019-0120-4 31687474 PMC6820995

[17] Li H., et al., The Role of ARV Associated Adverse Drug Reactions in Influencing Adherence Among HIV-Infected Individuals: A Systematic Review and Qualitative Meta-Synthesis. AIDS Behav, 2017. 21(2): p. 341–351. 10.1007/s10461-016-1545-0 27613645 PMC5290204

[18] Waal de R., et al., Routine data underestimates the incidence of first-line antiretroviral drug discontinuations due to adverse drug reactions: Observational study in two South African cohorts. PLoS One, 2018. 13(9): p. e0203530. 10.1371/journal.pone.0203530 30183766 PMC6124775

[19] Prosperi M.C.F., et al., Predictors of first-line antiretroviral therapy discontinuation due to drug-related adverse events in HIV-infected patients: a retrospective cohort study. BMC infectious diseases, 2012. 12(1): p. 296–296. 10.1186/1471-2334-12-296 23145925 PMC3519703

[20] Angamo M.T., et al., Mortality from adverse drug reaction-related hospitalizations in south-west Ethiopia: A cross-sectional study. J Clin Pharm Ther, 2018. 43(6): p. 790–798. 10.1111/jcpt.12702 29722039

[21] Angamo M.T., et al., Predictors of adverse drug reaction-related hospitalisation in Southwest Ethiopia: A prospective cross-sectional study. PLoS One, 2017. 12(10): p. e0186631. 10.1371/journal.pone.0186631 29036230 PMC5643118

[22] Tetteh R.A., et al., Association Between the Occurrence of Adverse Drug Events and Modification of First-Line Highly Active Antiretroviral Therapy in Ghanaian HIV Patients. Drug Saf, 2016. 39(11): p. 1139–1149. 10.1007/s40264-016-0460-7 27638659 PMC5045837

[23] Mouton J.P., et al., Adverse Drug Reactions Causing Admission to Medical Wards: A Cross-Sectional Survey at 4 Hospitals in South Africa. Medicine (Baltimore), 2016. 95(19): p. e3437. 10.1097/MD.0000000000003437 27175644 PMC4902486

[24] NATIONS, U., TRANSFORMING OUR WORLD: THE 2030 AGENDA FOR SUSTAINABLE DEVELOPMENT. 2015/2016.

[25] Gebrezgabher B.B., et al., Determinants to antiretroviral treatment non-adherence among adult HIV/AIDS patients in northern Ethiopia. AIDS Res Ther, 2017. 14: p. 16. 10.1186/s12981-017-0143-1 28331527 PMC5359813

[26] Ataro Z., et al., Magnitude and causes of first-line antiretroviral therapy regimen changes among HIV patients in Ethiopia: a systematic review and meta-analysis. BMC Pharmacol Toxicol, 2019. 20(1): p. 63. 10.1186/s40360-019-0361-3 31675986 PMC6824137

[27] Gelaw Y.K., et al., Coping Strategies for Adverse Effects of Antiretroviral Therapy among Adult HIV Patients Attending University of Gondar Referral Hospital, Gondar, Northwest Ethiopia: A Cross-Sectional Study. AIDS research and treatment, 2018. 2018: p. 1879198–10. 10.1155/2018/1879198 30631595 PMC6304486

[28] Food, M.a.H.A.a.C.A.o.E., *Guideline for Adverse Drug Events Monitoring (Pharmacovigilance)* Third Edition. 2014.

[29] Weldegebreal F., Mitiku H., and Teklemariam Z., Magnitude of adverse drug reaction and associated factors among HIV-infected adults on antiretroviral therapy in Hiwot Fana specialized university hospital, eastern Ethiopia. Pan Afr Med J, 2016. 24: p. 255. 10.11604/pamj.2016.24.255.8356 27800108 PMC5075466

[30] Eluwa G.I., et al., Adverse drug reactions to antiretroviral therapy (ARVs): incidence, type and risk factors in Nigeria. BMC Clin Pharmacol, 2012. 12: p. 7. 10.1186/1472-6904-12-7 22369677 PMC3317861

[31] Hasan S.S., et al., *Patient-reported adverse drug reactions and drug-drug interactions*: *a cross-sectional study on Malaysian HIV/AIDS patients*. Med Princ Pract, 2011. 20(3): p. 265–70. 10.1159/000321274 21454998

[32] Kindie E., Alamrew Anteneh Z., and Worku E., Time to development of adverse drug reactions and associated factors among adult HIV positive patients on antiretroviral treatment in Bahir Dar City, Northwest Ethiopia. PLoS One, 2017. 12(12): p. e0189322. 10.1371/journal.pone.0189322 29267292 PMC5739414

[33] Gudina E.K., et al., Magnitude of Antiretroviral Drug Toxicity in Adult HIV Patients in Ethiopia: A cohort study at seven teaching hospitals. Ethiop J Health Sci, 2017. 27(Suppl 1): p. 39–52. 10.4314/ejhs.v27i1.5s 28465652 PMC5402801

[34] Hagos L., Fessehaye S., and Anand I.S., Nature and prevalence of adverse drug reaction of antiretroviral medications in Halibet National Referral Hospital: a retrospective study. BMC pharmacology & toxicology, 2019. 20(1): p. 24. 10.1186/s40360-019-0307-9 31060603 PMC6503428

[35] Delicio A.M., et al., Adverse effects of antiretroviral therapy in pregnant women infected with HIV in Brazil from 2000 to 2015: a cohort study. BMC Infect Dis, 2018. 18(1): p. 485. 10.1186/s12879-018-3397-x 30261855 PMC6161436

[36] WHO, *Consolidated guidelines on the use of antiretroviral drugs for treating and preventing HIV infection*: *recommendations for a public health approach*. 2016: Geneva, sewitherland. 27466667

[37] Asfaw L. and Enqueselassie F., Time to Major Adverse Reactions of Anti-Retroviral Drugs and its Predictors among Cohort of Patients Receiving Antiretroviral Therapy in Hosanna Hospital, Hosanna, Ethiopia: Retrospective Cohort Study. International Journal of Virology and AIDS, 2018. 5.

[38] Shet A., et al., Influence of adverse drug reactions on treatment success: prospective cohort analysis of HIV-infected individuals initiating first-line antiretroviral therapy in India. PLoS One, 2014. 9(3): p. e91028. 10.1371/journal.pone.0091028 24614165 PMC3948746

[39] O. D. H. K. v.d.B. L. M. J. L. L.C., Adverse Drug Reactions Among Patients Initiating Second‑Line Antiretroviral Therapy in South Africa. Drug Sefty, 2018. 41: p. 1343–1353. 10.1007/s40264-018-0698-3 PMC6223700 30043384

[40] Lartey M., et al., Adverse drug reactions to antiretroviral therapy during the early art period at a tertiary hospital in Ghana. Pan Afr Med J, 2014. 18: p. 25. 10.11604/pamj.2014.18.25.3886 25368714 PMC4214560

[41] d Pádua C.A.M., et al., High incidence of adverse reactions to initial antiretroviral therapy in Brazil. Brazilian journal of medical and biological research = Revista brasileira de pesquisas medicas e biologicas, 2006. 39(4): p. 495–505. 10.1590/s0100-879x2006000400010 16612473

[42] Mudigubba M., et al., A systematic review of risk factors of adverse drug reactions in hospitalized patients. Asian Journal of Pharmaceutical and Clinical Research, 2018. 11: p. 25.

[43] Zucker I. and Prendergast B.J., Sex differences in pharmacokinetics predict adverse drug reactions in women. Biol Sex Differ, 2020. 11(1): p. 32. 10.1186/s13293-020-00308-5 32503637 PMC7275616

[44] Abah I.O., et al., Incidence and predictors of adverse drug events in an African cohort of HIV-infected adults treated with efavirenz. Germs, 2015. 5(3): p. 83–91. 26405676 10.11599/germs.2015.1075 PMC4570838

[45] Mwesigire Doris Mutabazi, W A.W., Martin Faith, Katamba Achilles and Seeley Janet, Quality of life in patients treated withfirst-line antiretroviral therapy containingnevirapine or efavirenz in Uganda: aprospective non-randomized study. BMC 2015. 10.1186/s12913-015-0959-0 26216221 PMC4517416

